# Supplementary material for: Machine learning for cluster analysis of localization microscopy data
Source: Nat Commun. 2020 Mar 20;11:1493. doi: 10.1038/s41467-020-15293-x (PMC7083906; doi:10.1038/s41467-020-15293-x)
Supplement: Supplementary file 1 — Supplementary Information [file 41467_2020_15293_MOESM1_ESM.pdf]

Supplementary Information

Machine-learning for cluster analysis of localization microscopy data

Williamson *et al*.

Supplementary Figures

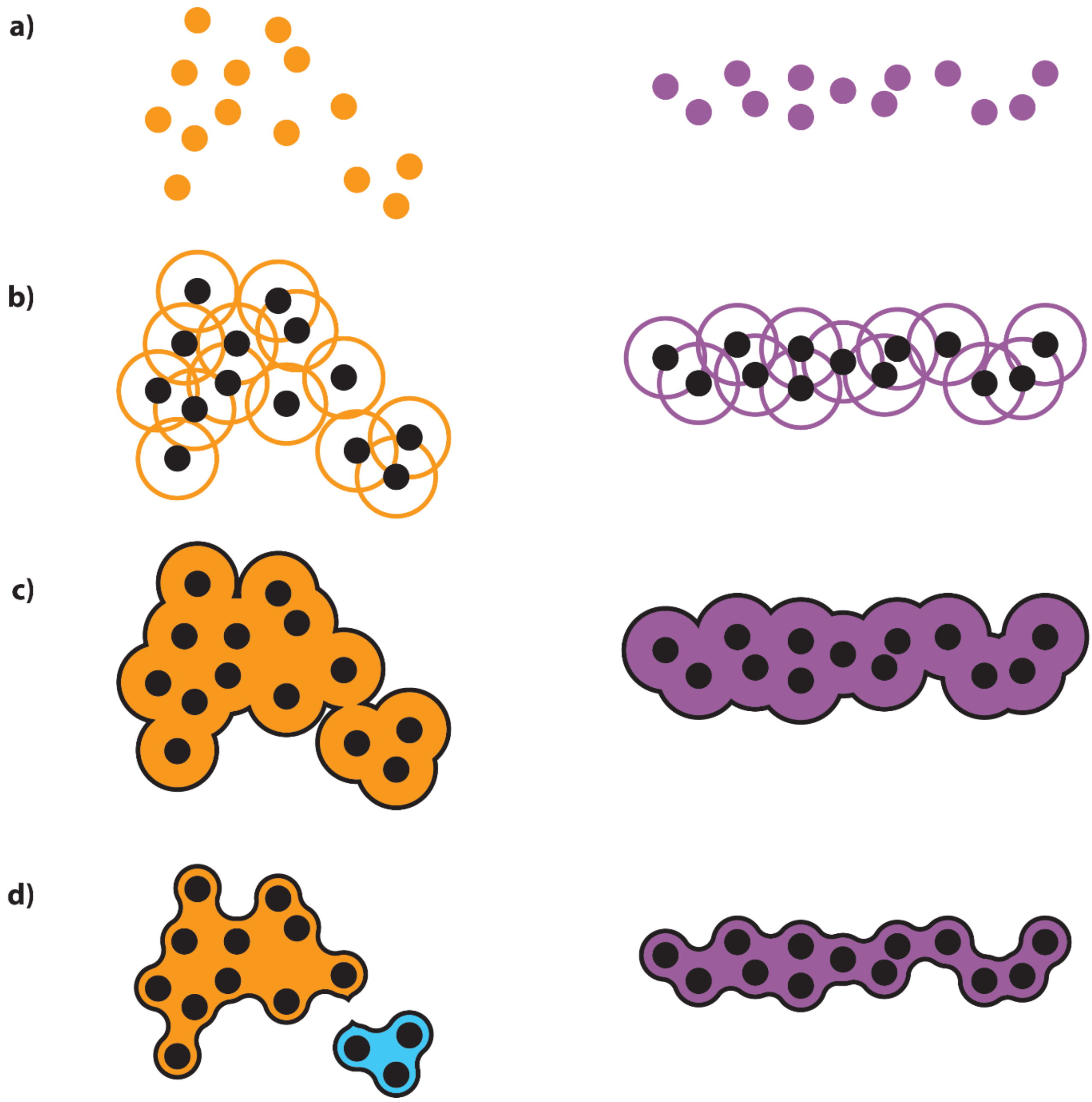

Supplementary Figure 1. Formation of cluster shapes. After points are assigned to specific clusters (a), all points from the same cluster are isolated and each point is dilated by a certain radius (b). Taking the union of all overlapping shapes describes the shape of the cluster (c). This shape is then eroded to return a new shape that more tightly fits the points (d). Some points may become separated from the original cluster; this may yield a new cluster, or the point can be reclassified.

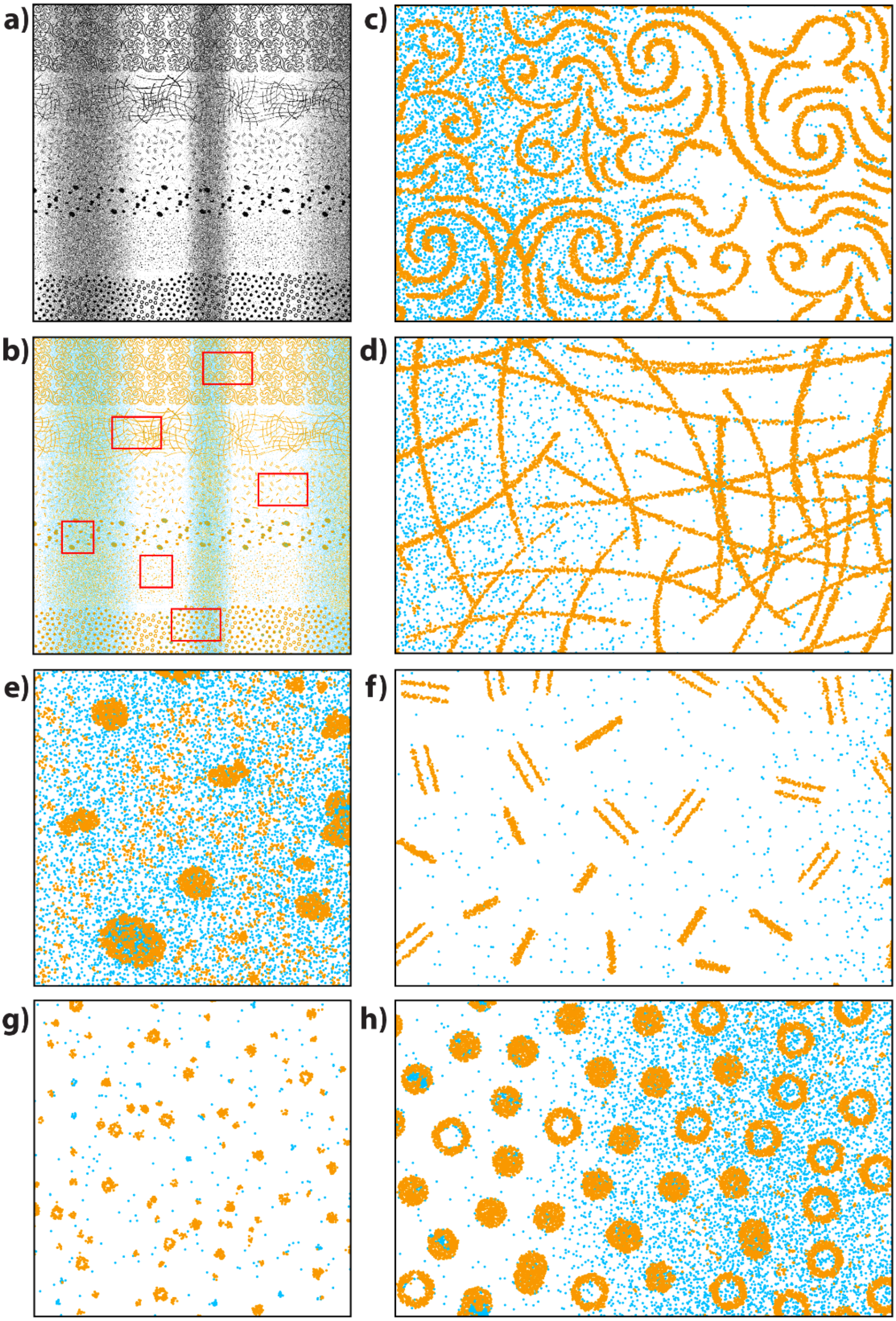

Supplementary Figure 2. A challenging dataset containing structures and variable background densities which were not encountered during training. An intensity mask image was used with ThunderSTORM's data generator function to create a point patterned dataset (a) which was processed with CAML Model 87B144 (b, orange points are clustered, and blue are non-clustered). The model was able to classify points from fibrous clusters (c, d) even across sharp changes in non-clustered point density. Very large and dense clusters (e) were more challenging. Clustered points were correctly identified in highly elongated structures (f) as well as small ring-shaped clusters and

clusters with Gaussian density profiles (g). Points in large, dense and ring-shaped clusters were also identified (h).

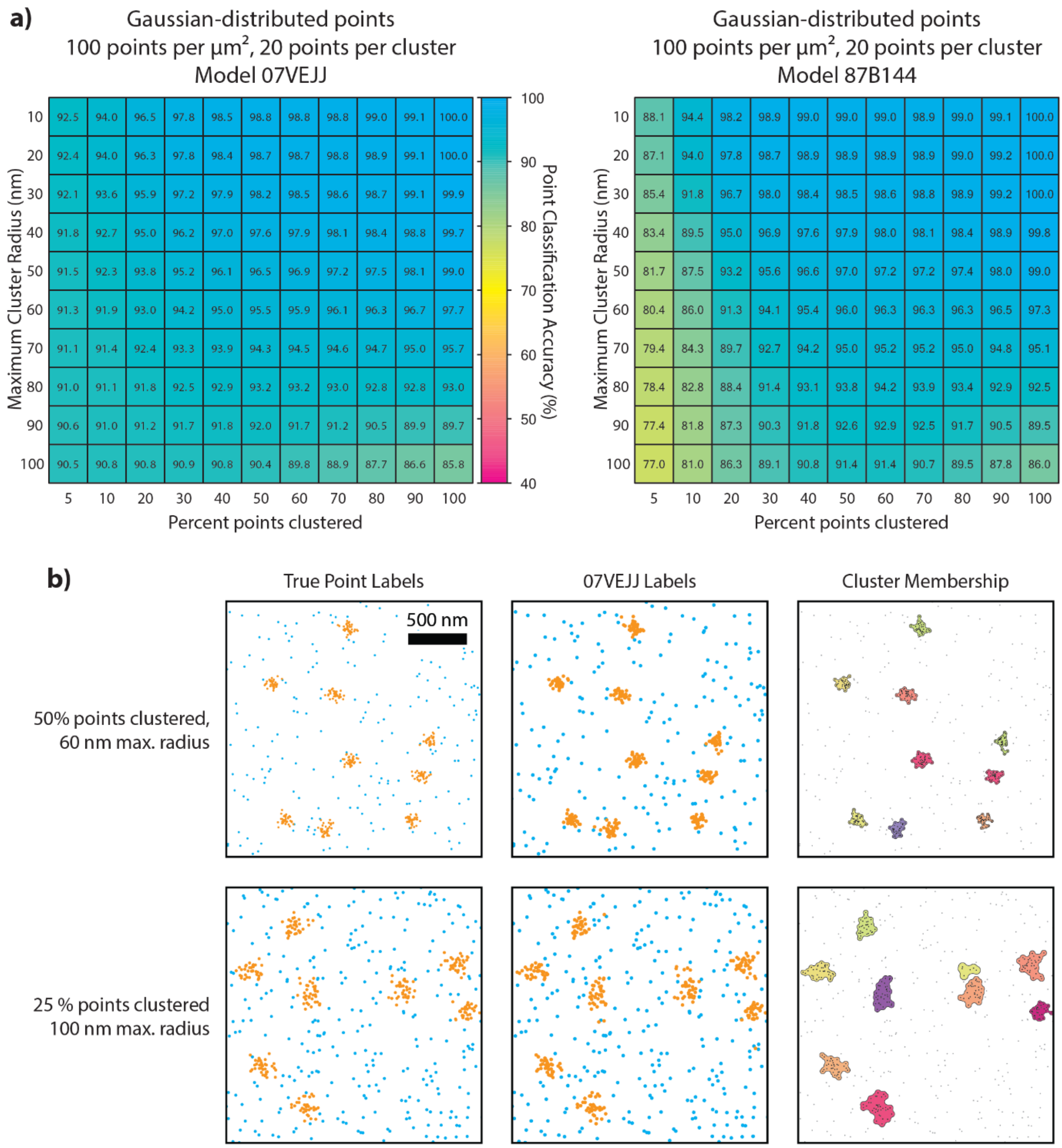

Supplementary Figure 3. Performance on cluster scenarios where clustered points are distributed by a Gaussian distribution. Cluster scenarios were generated and evaluated by Model 07VEJJ (a) or Model 87B144 (b) both of which returned over 90% classification accuracy. Example regions from two cluster scenarios are shown in c) comparing the true point labels (clustered or not), the model-assigned point labels (for 07VEJJ using 100 nearest neighbors), and finally the cluster membership. Data are mean values from three replicates.

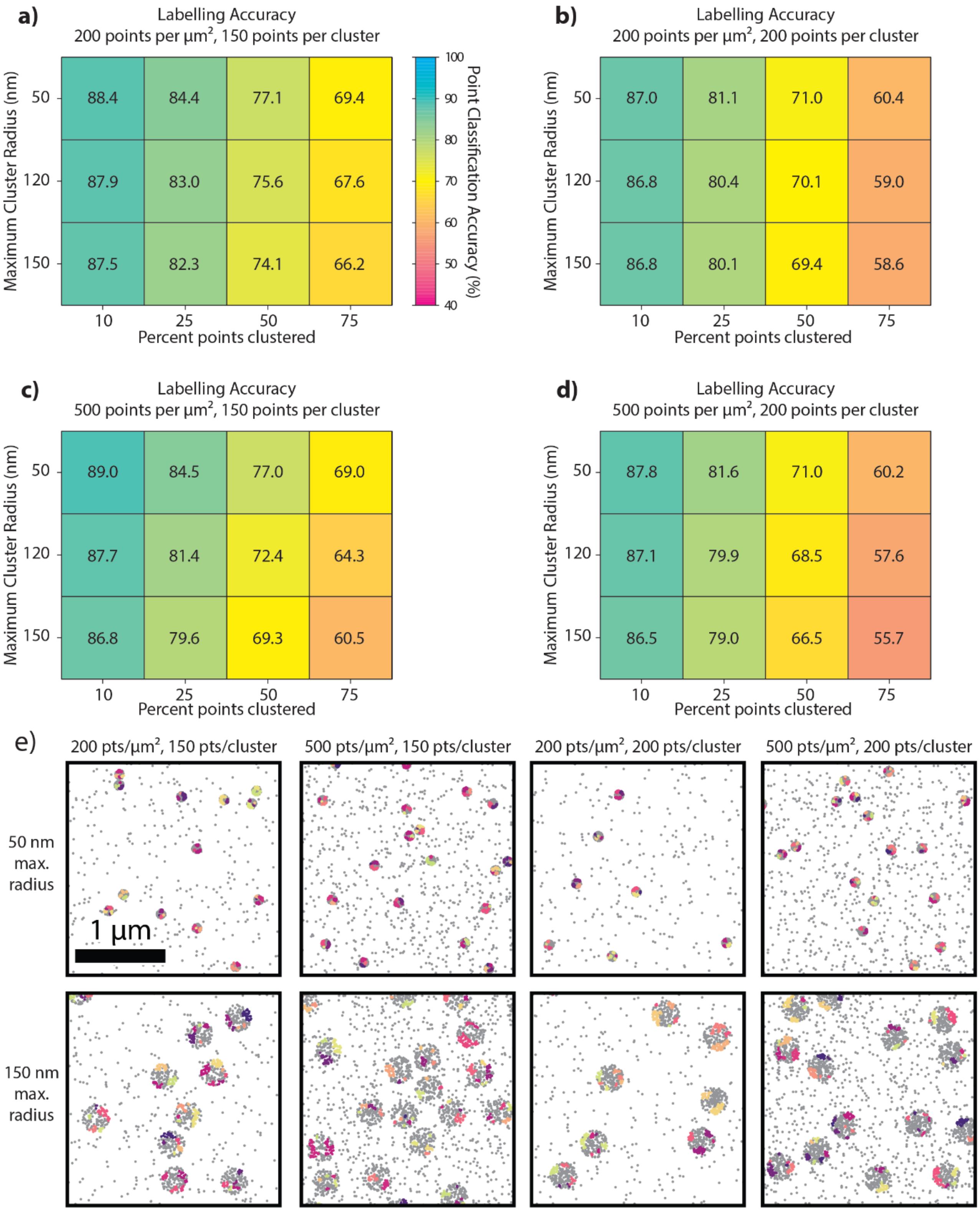

Supplementary Figure 4. Effect of exceeding a model's input capacity. Cluster scenarios describing 150 (a, c) or 200 (b, d) points per cluster were evaluated with Model 07VEJJ. Example regions from these cluster scenarios (all with 75% of points clustered) demonstrate how the model, which operates on input from 100 nearest neighbors, fails to correctly identify clustered points resulting in poor cluster segmentation (e). Data in a)–d) are mean values from three replicates.

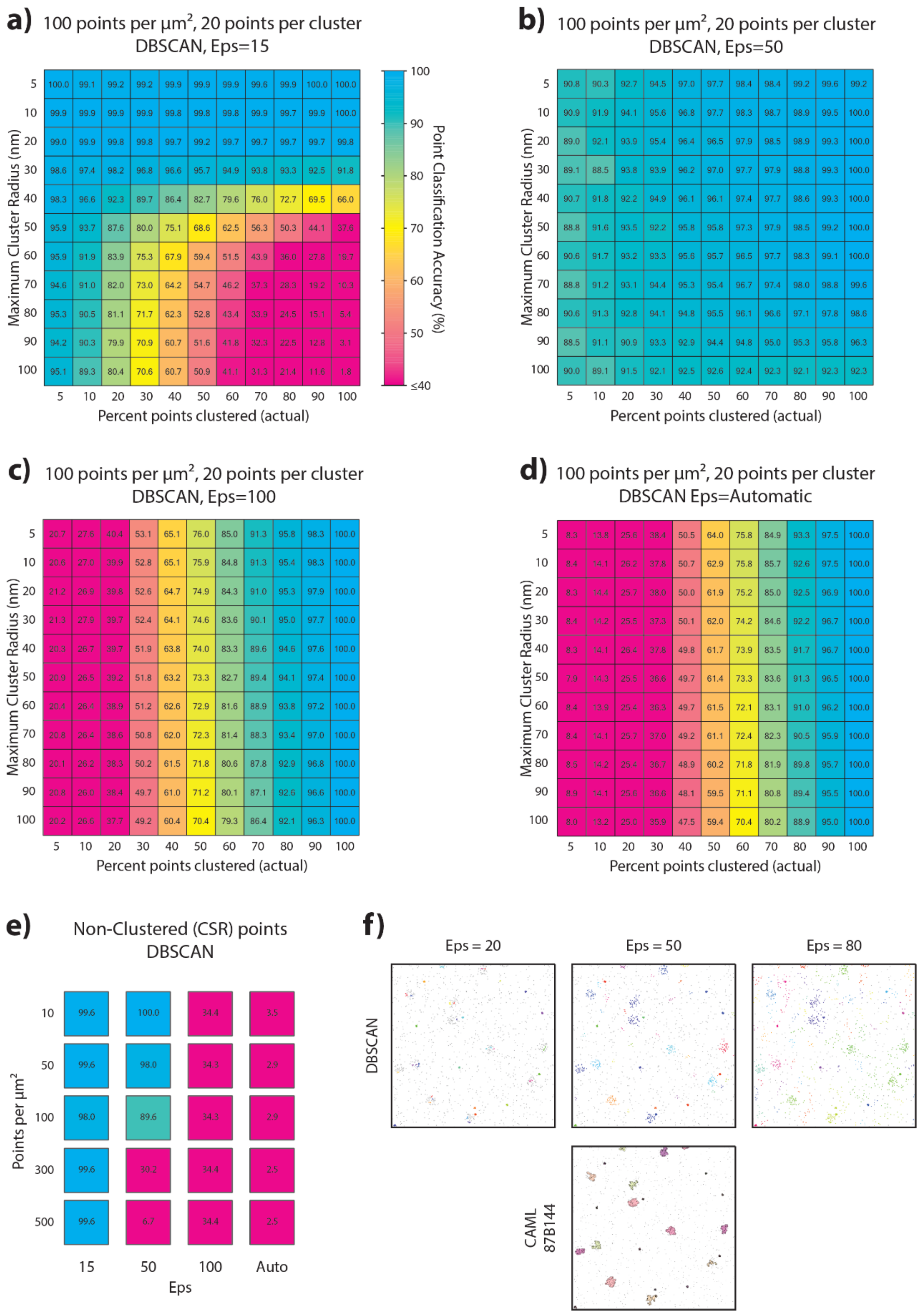

Supplementary Figure 5. Classification accuracy of simulated clustered data (as in **Error! Reference source not found.**a, g) assessed with DBSCAN using various values for the 'epsilon' parameter: (a) Eps=15, (b) Eps=50, (c) Eps=100, and (d) Eps=Automatic. The same parameters were also used to assess performance on non-clustered, spatially random data (e). There were n = 10 images for each clustering scenario. Parameter selection in DBSCAN becomes especially sensitive when there are multiple types of clusters, e.g. small, dense clusters among large, sparse clusters as in (f). Data are mean values from ten replicates.

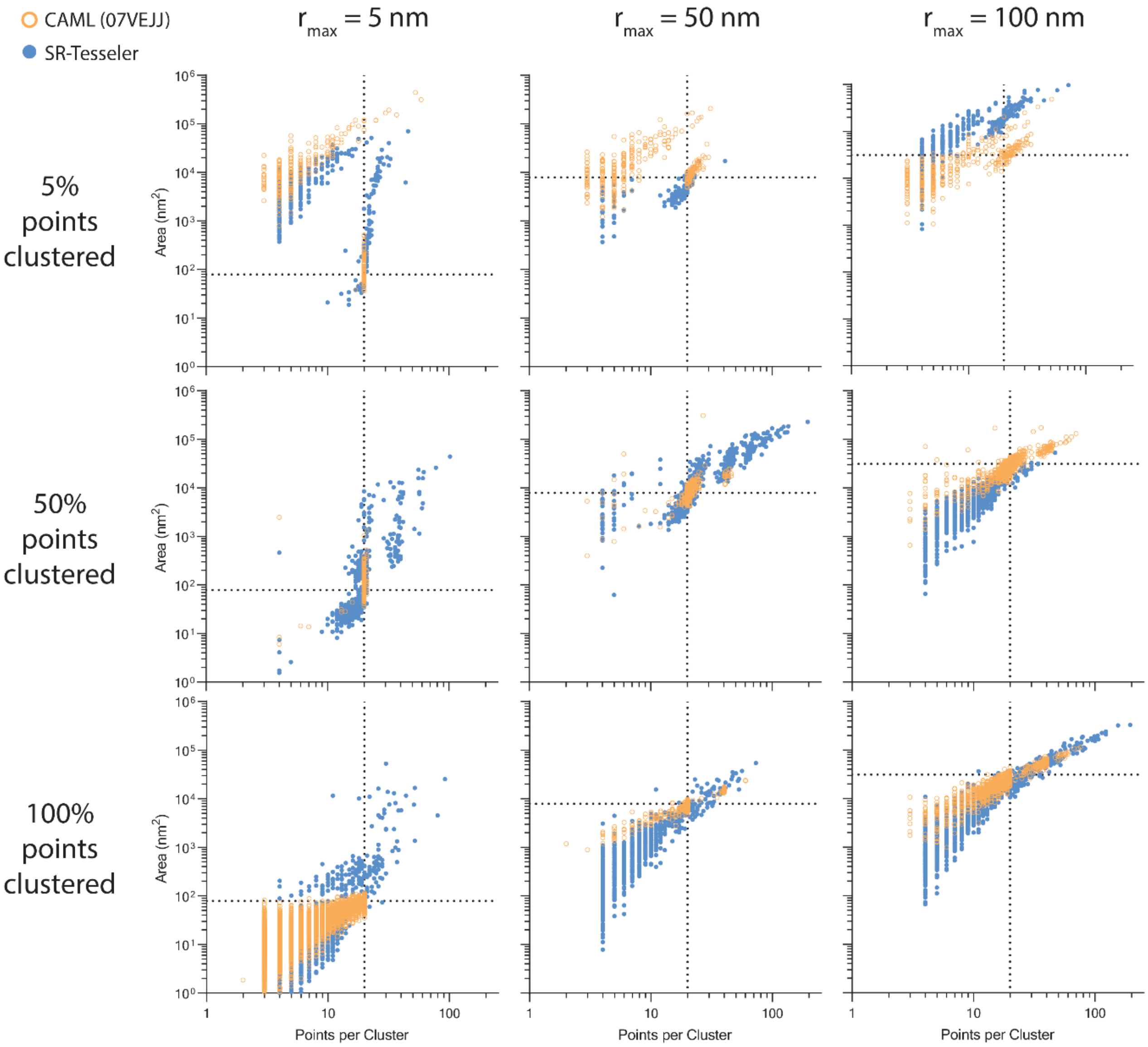

Supplementary Figure 6. Area and points-per-cluster from nine simulated clustering scenarios (100 points per μm^2 and 20 points per cluster; the same data as used in **Error! Reference source not found.**a) assessed with SR-Tesseler (blue solid-circles) and CAML (Model 07VEJJ, orange open-circles). The target values for each cluster scenario are marked by dashed lines; data are from a single simulated image from each cluster scenario.

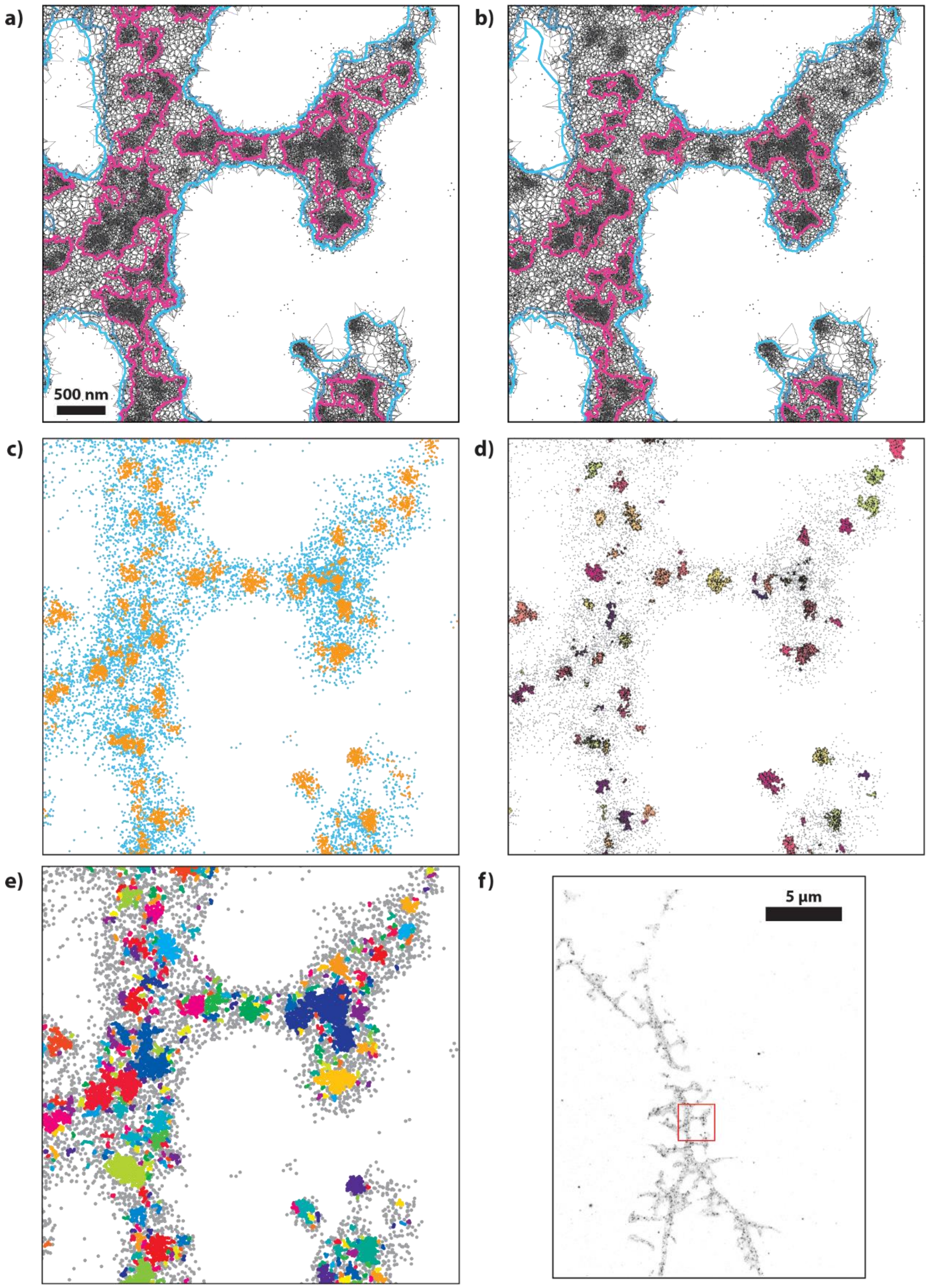

Supplementary Figure 7. Comparison of CAML to SR-Tesseler and DBSCAN for PALM data of GRIA1 (GluA1) fused to mEos2 in neurons. Analysis with SR-Tesseler with two different density factor (δ) parameters (a, δ = 0.75 and b, δ = 1.00; object outlines are blue and cluster outlines are magenta). Analysis of the same data using CAML (c, Model 87B144; clustered points are orange and non-clustered points are blue) with cluster segmentation (d). The same data were also processed with DBSCAN (e, minPts = 3, Eps = 11.8). Panel f) shows the field where the enlarged region (in red) is located.

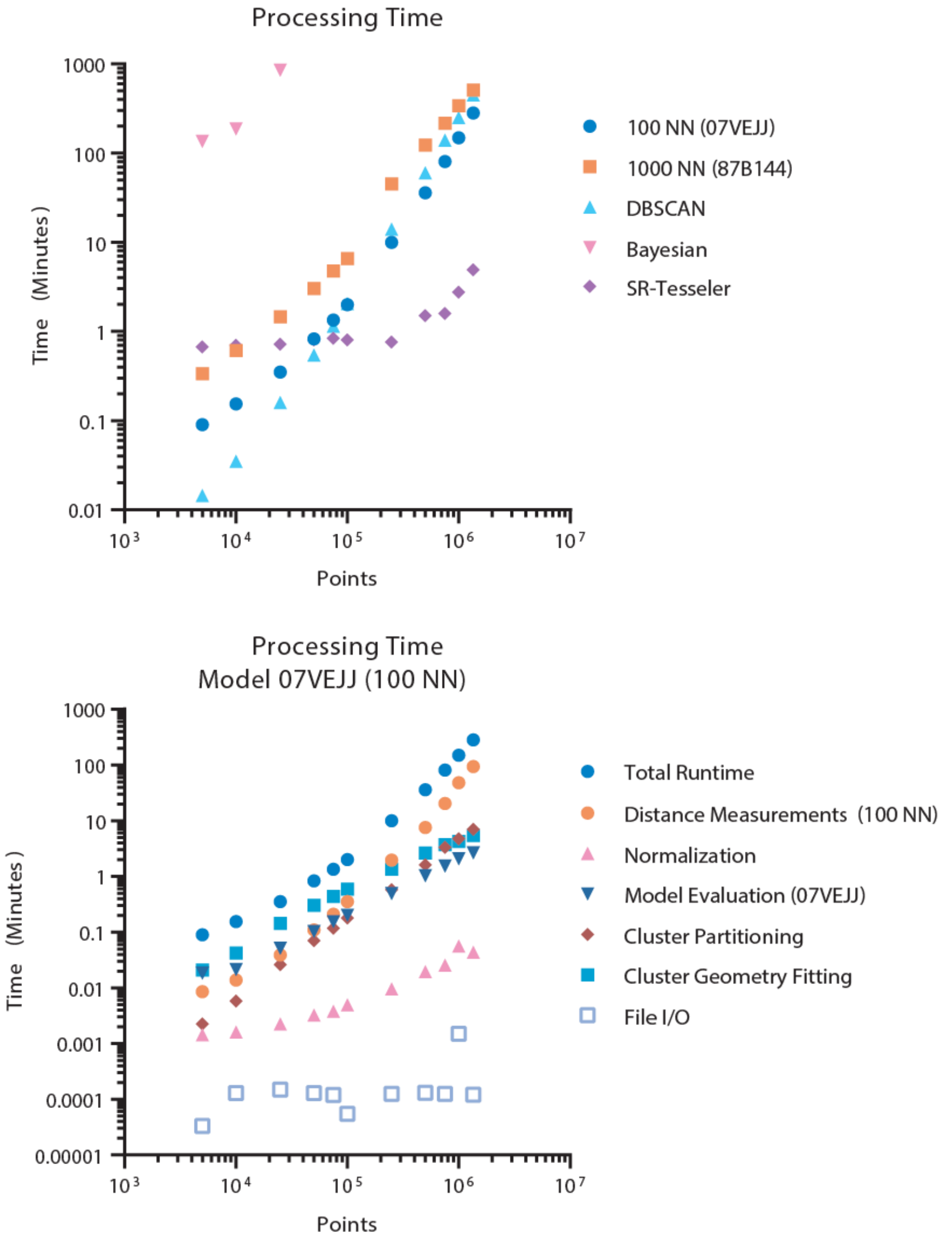

Supplementary Figure 8. Comparison of processing times for different numbers of points: a) CAML models 07VEJJ (dark blue filled circles) and 87B144 (orange squares), DBSCAN (light blue up-triangles), Bayesian (pink down-triangles), and SR-Tesseler (purple diamonds). CAML times are the sum of all processing stages for evaluation of data with a trained model, i.e. distance measurements, model classification, cluster segmentation, forming cluster shapes, and data export. Processing times for DBSCAN include file load, cluster segmentation, and data export; DBSCAN does not perform cluster shape-forming. Timings for Bayesian include file load, full Bayesian analysis, cluster segmentation, cluster shape formation (approximated as the mean point distance from the centroid mean) and results export. Timings for SR-Tesseler include file load, tessellation on all points (without the 'detection cleaning' step), and interactive cluster segmentation. A breakdown of processing times for specific operations when using model 07VEJJ is shown in b) where the total runtime (dark blue filled circles) includes distance measurements (orange circles), data normalization (pink triangles), evaluation with the model (dark blue triangles), partitioning of clustered points into like-clusters (red diamonds), fitting of shapes around like-clustered points (blue squares) and system file operations (loading & saving data, grey open squares). Assessment was performed on an Intel Core i7-4930K CPU (using up to 10 cores where applicable) and NVIDIA GTX 750 Ti GPU. Results are from a single test image per condition, each condition's input image was derived as a random subset of points from the largest condition, an image with 1.35 million points.

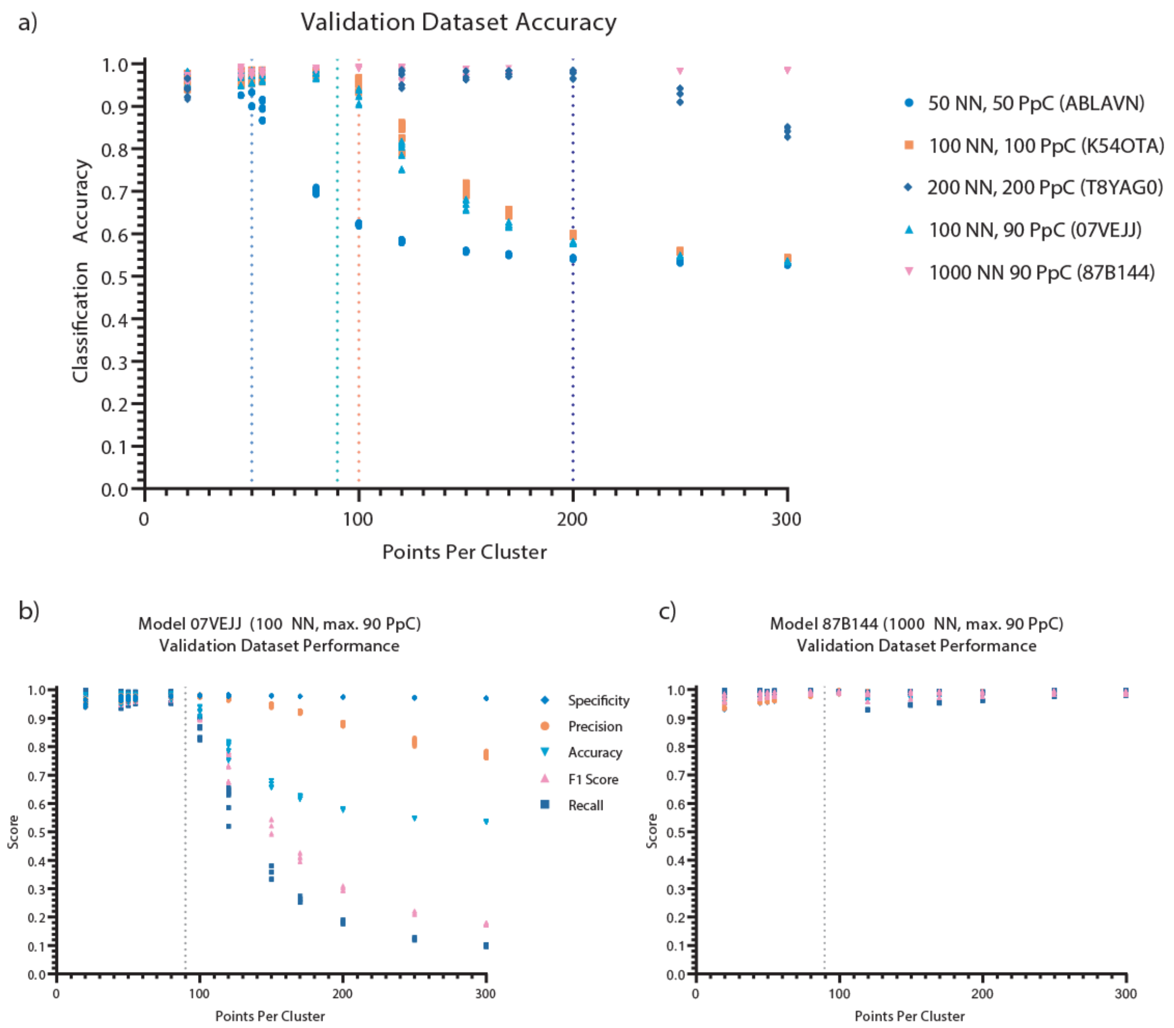

Supplementary Figure 9. The effect of a model's input size (i.e. number of near-neighbor distances) and training data (maximum points per cluster encountered during training) on accuracy. In a), various models were configured with different input windows (using 50, 100, 200, 1000 NN values) and training data with different maximum points-per cluster and then given an evaluation dataset feature clusters that contained points beyond the model's experience. Models are only accurate when the clusters contain fewer points than the model's input window; note Model 87B144 was trained on clusters up to 90 points-per-cluster but, with an input window of 1000 NN, is capable of high accuracy when given clusters containing many more points. Plots for additional performance metrics of models 07VEJJ (b) and 87B144 (c) show that the performance deficit results in a failure to correctly identify clustered points (drop in recall but not specificity); points in the center of a dense cluster will be most greatly affected by misclassification, as in Supplementary Figure 4.

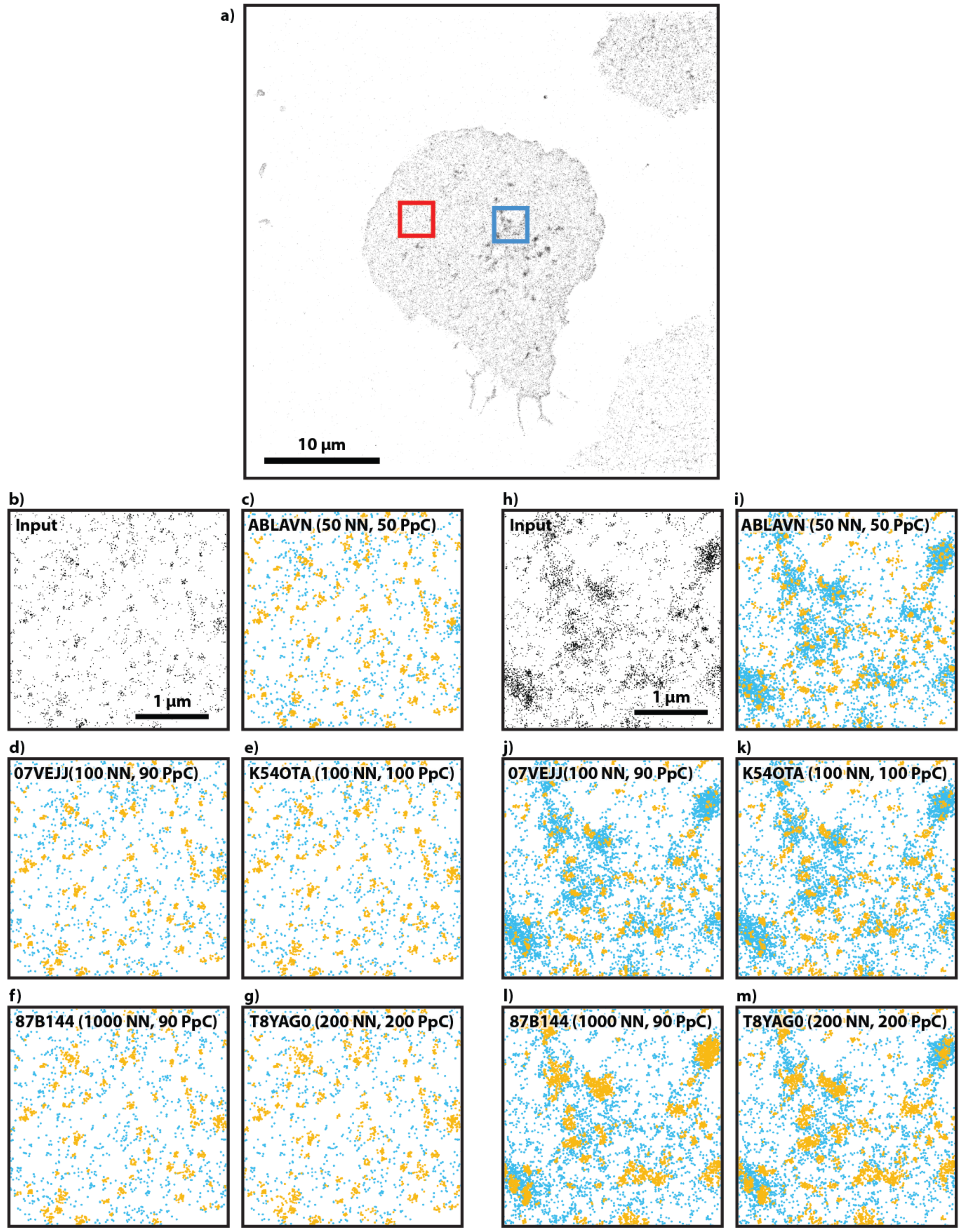

Supplementary Figure 10. The effect of a model's input size (i.e. number of near-neighbor distances) and training data (maximum points per cluster encountered during training) on cluster identification in 'real' data. a) LAT-mEos3.2 imaged by PALM in a Jurkat cell synapse formed against antibody-coated glass. The whole image was analyzed by the models described in Supplementary Figure 9. Two 3 × 3 µm regions are shown below for peripheral (red box, b) and central (blue box, h) areas of the synapse. Data in these regions for each model are shown in (c-g; peripheral) and (i-m; central) with 'clustered' points in orange and 'non-clustered' points in blue. Models all feature the same arrangement of 12 layers, as described for model 07VEJJ.

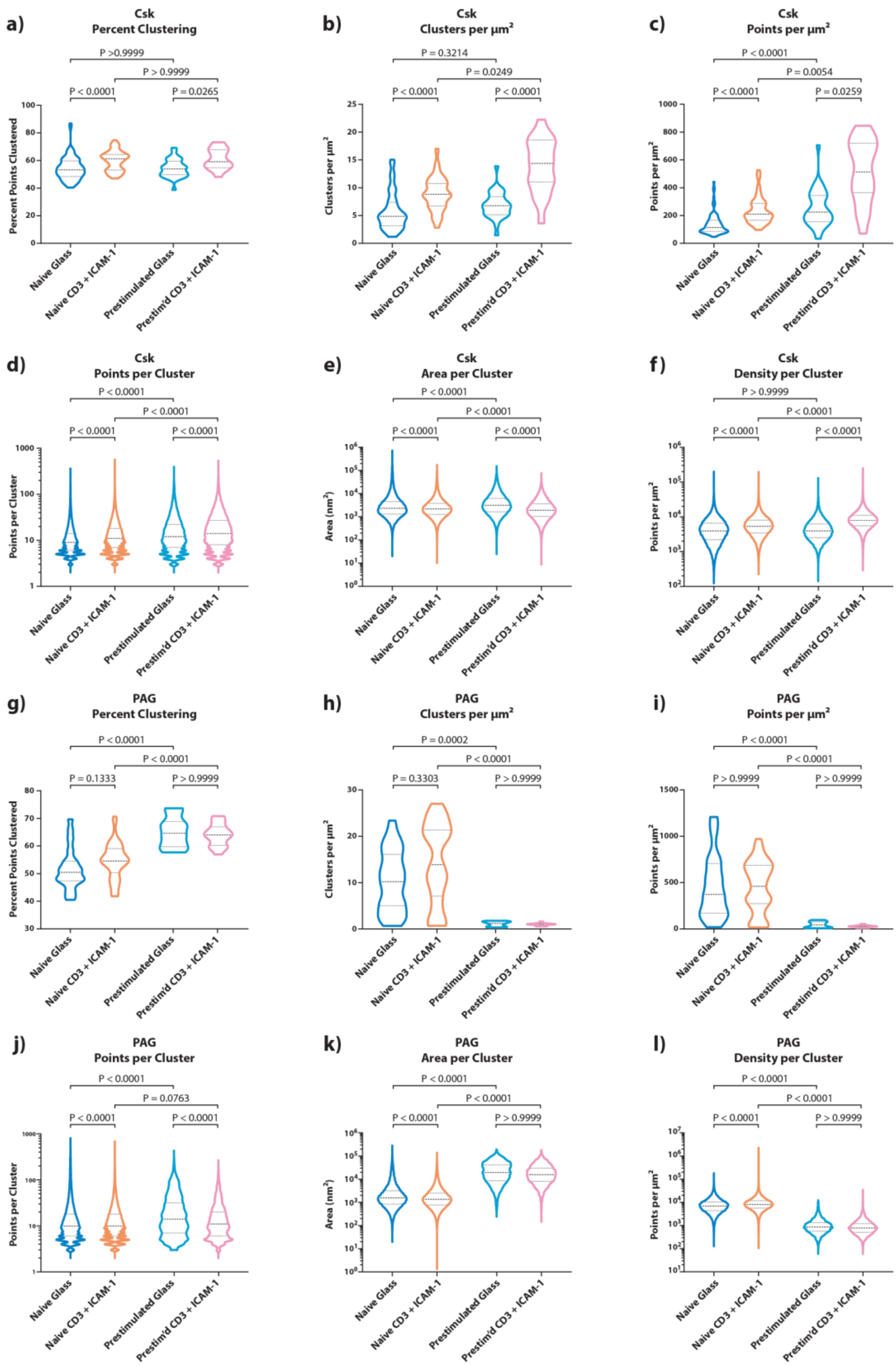

Supplementary Figure 11. Descriptions of clustering for Csk (a-f) and PAG (g-l) after assessment by Model 87B144. Data in panels a-c and g-i are from individual cells. Data in panels d-f and j-l are from all identified clusters. Data are presented as median values ± quartiles and are from three independent experiments; Csk, Naïve - Glass: 85 cells (23672 clusters), Csk, Naïve - CD3 + ICAM-1: 71 cells (64809 clusters), Csk, Pre-stimulated - Glass: 30 cells (34954 clusters), Csk, Pre-stimulated - CD3 + ICAM-1: 19 cells (58476 clusters), PAG, Naïve - Glass: 53 cells (31813 clusters), PAG, Naïve - CD3 + ICAM-1: 65 cells (98455 clusters), PAG, Pre-stimulated - Glass: 14 cells (3156 clusters), PAG, Pre-stimulated - CD3 + ICAM-1: 18 cells (4309 clusters). Pairwise comparisons were performed by non-parametric Kruskal-Wallis and Dunn's multiple comparison tests. Summary statistics are presented in Supplementary Tables 2 and 3.

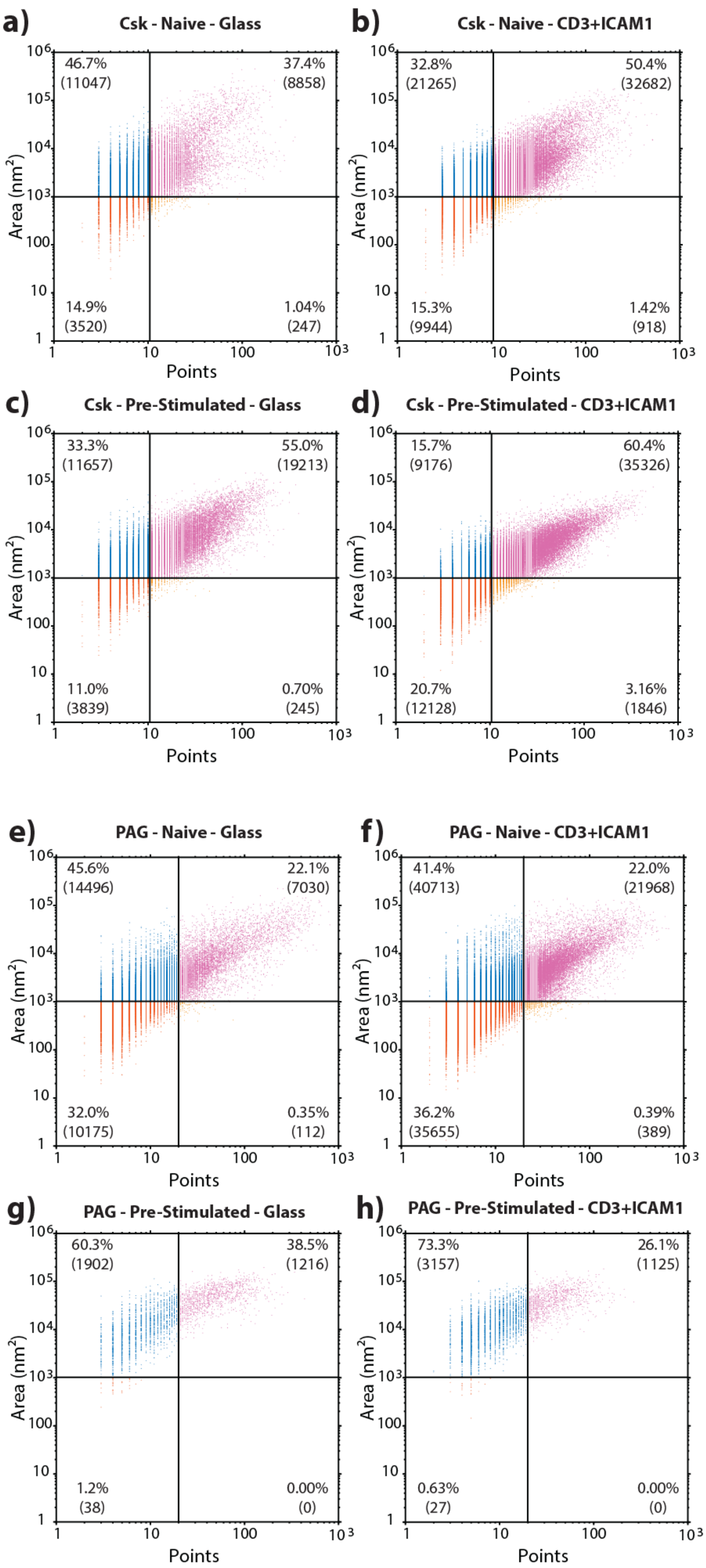

Supplementary Figure 12. Scatterplots of total points and area of individual Csk (a-d) and PAG (e-h) clusters identified by Model 87B144. Quadrants demonstrate how cluster populations shift between different cell types (naïve or pre-stimulated) and different environmental conditions (glass, non-stimulated or CD3+ICAM, stimulated). The percent (and number) of clusters are given in each quadrant. Data are from three independent experiments.

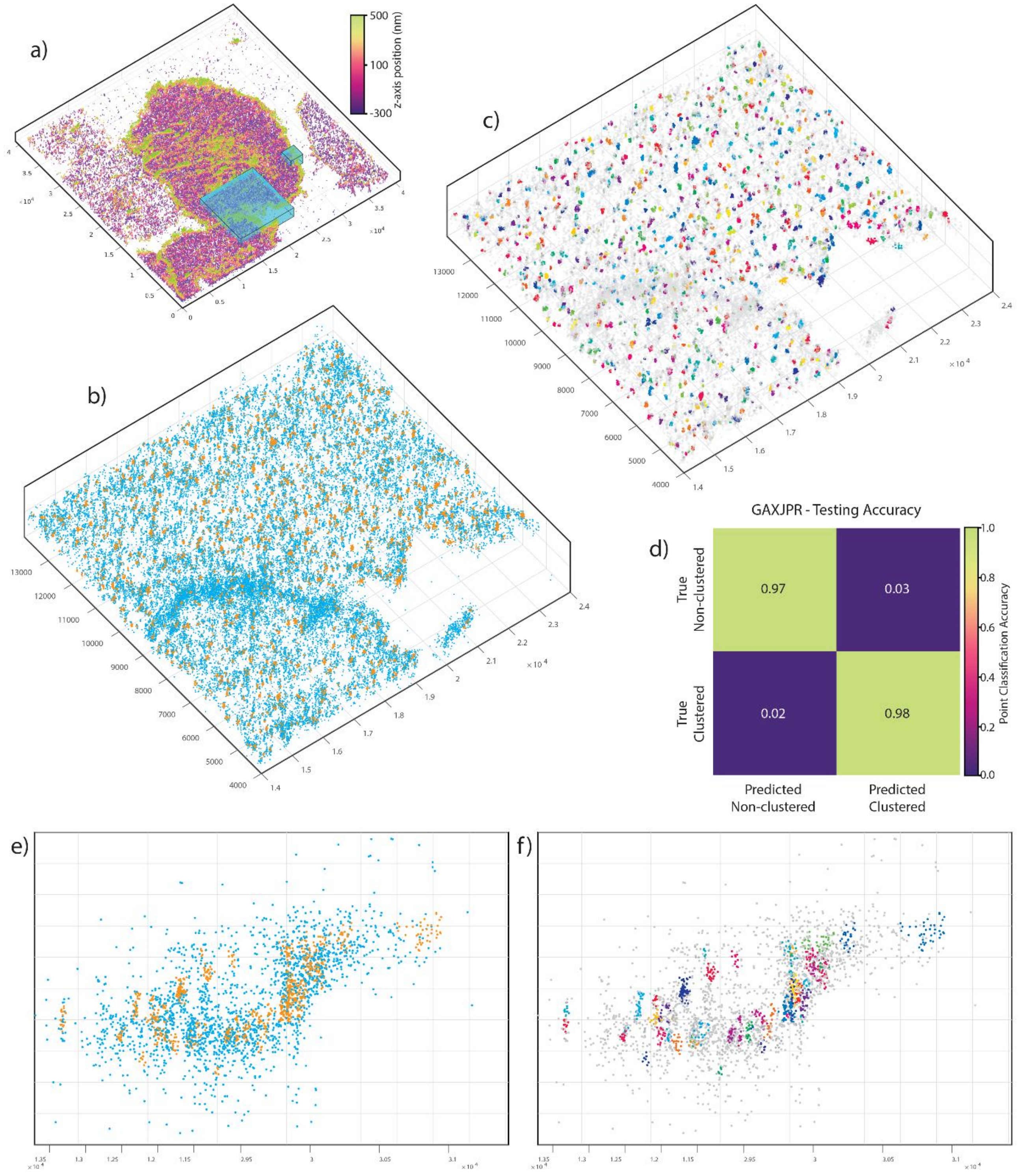

Supplementary Figure 13. Extension of the method to three-dimensional data. A PALM image of Jurkat T-like cells (expressing LAT-mEos3.2 and induced to form synapses on antibody-coated glass) is evaluated by a model trained on 3D simulated data of spherical clusters. An overview of the image is shown in a) with points colored by axial position and enlarged regions indicated by blue volumes. The larger region is shown in b) and c) with points colored by model classification label or cluster membership, respectively. The model's accuracy on testing data is shown in d). The smaller region from a) is shown in e) and f), rotated to show a projection of the membrane curvature at the edge of the cell. Clusters of LAT are identified both within the plasma membrane and in membrane-proximal vesicles. Note the non-isotropic localization of molecules in the axial direction does not prevent the identification of clustered points.

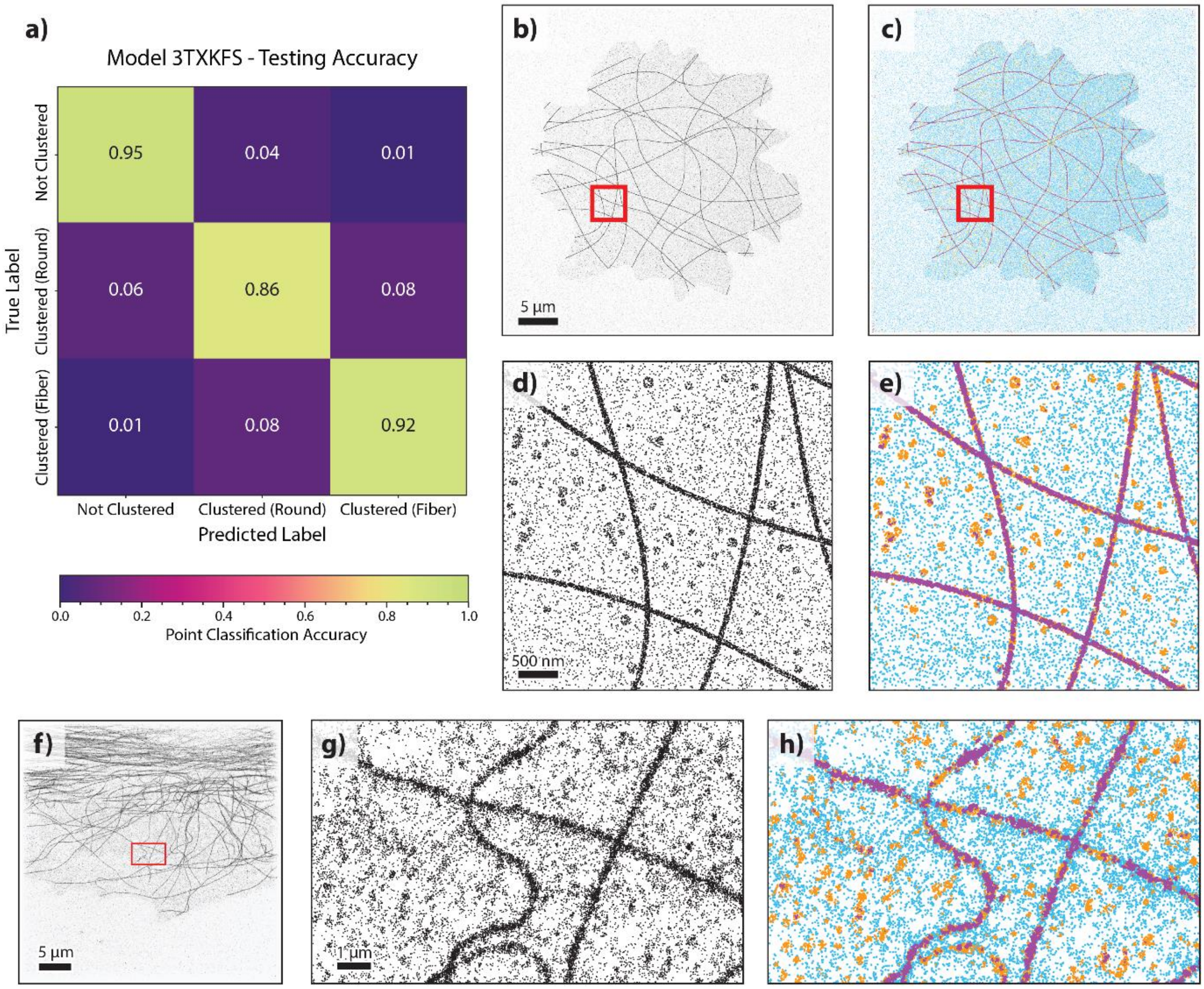

Supplementary Figure 14. Extension of the method to use multiple labels for classification of points. A new model was trained on simulated data (combined from data tables produced by ThunderSTORM's data generator function using a separate intensity mask for each type of point) where points were distributed in either round clusters, long filaments, or randomly in the space between the clustered points; performance on the testing dataset is shown in a). An equivalent simulated dataset (b and zoomed region d) was evaluated with the model and the points classified as one of the three types (c, e; blue: not clustered, orange: clustered (round), magenta: clustered (fibers). The model was also used to evaluate an image of microtubules (f, g, h) in HFF cells and separates points from intact microtubules (as fiber clusters), fragments of filaments (as round clusters) or as monomeric/non-specific (non-clustered) points.

Supplementary Methods

Simulated data generation

Each simulated dataset represented a defined, homogeneous clustering of points. Each clustering scenario was drawn from the combination of a range of clustering parameters describing the distribution of points within clusters and within the image field (Supplementary Note 1). Each generated dataset file contained a pair of *x* and *y* coordinates, arranged in columns, for each point. Simulated data included additional values indicating the unique cluster ID to which that point belonged, or zero to indicate a non-clustered point. The points were contained within a two dimensional 'field of view' comparable to that of an SMLM instrument (40 × 40 μm).

Within this field of view, a polygon was generated to represent the 'cell-like' boundary shape and within this shape, points were distributed according to a specified clustering scenario. For scenarios containing clustered points, the total number of clusters was determined and a set of coordinates, each representing the seed-point for a cluster, was randomly distributed within the image field. Seed-points which fell outside of the cell-boundary shape or which were inside but located too close to either the cell outline (i.e. within the designated maximum cluster radius) or to another cluster seed (i.e. within the twice the maximum cluster radius) were randomly re-positioned until all the required seed points were satisfactorily located.

Clustered points were then distributed around each cluster-seed according to that scenario's specifications and in a uniform distribution to form disc-shaped (hard edged) clusters. The number of non-clustered points was determined, and these points were uniformly distributed within the cell-shape. In order to preserve the clustering properties of the final dataset, any non-clustered points which were placed within the maximum radius (according to the cluster scenario) of a cluster seed were relocated. This ensures that the 'clusters' always contained the specified number of points, at the specified density. Finally, a delimited text file was saved containing the coordinates of all points, the cluster label for each point (0 for non-clustered and 1 for clustered), and a cluster ID value indicating to which specific cluster a point belonged. This process was repeated for each replicate of the cluster scenario and for all cluster scenarios; no two generated images shared either the same 'cell outline' or the same *xy* arrangement of cluster seeds or points. Simulated data were also generated for performance testing of trained models.

For Supplementary Figures 2 and 14, data were simulated using ThunderSTORM's data generator function. Here, a 16-bit intensity mask determines the spatial density of generated points within the image and allows for the creation of complex structures and density gradients. As is, the generator does not have any ground-truth information on the clustering status of the generated points. To include this information (as in Supplementary Figure 14), intensity masks must be created for each type of point. To prevent points of different types appearing in the same place (e.g. to avoid adding 'non-clustered' points to areas covered by 'clustered' points) then pixels within the masks need to be mutually exclusive. After data generation, the points for that mask are so labelled. The separate datasets for each label are then combined into a single dataset and the order of the points is randomized. This final dataset contains points in all the desired structures plus the ground truth labels for each point and can then be used for training or performance testing.

Data preparation

Here, we take the list of point coordinates and for each point determine the Euclidean distances to the specified number of near neighbors. A unique identifier for each point in the set is also assigned and saved with the sequence of distances to a memory-mapped file. This is the most resource-heavy aspect of the method as it can require large amounts of storage space if many images are to be analyzed, e.g. the distances for 1 million points to their nearest 1,000 neighbors (stored as 64-bit floating-point values) would require some 8 gigabytes of storage space.

Large datasets can become a problem for analysis methods which attempt to operate upon all points at the same time, exhausting the available computational resources. Strategies to effectively manage larger datasets can include acquiring more powerful computing resources or breaking a large dataset into manageable chunks and recombining the results after processing is complete. Some approaches discard data in order to reduce the demand on resources while other strategies avoid the holistic approach and focus on smaller, local subsets of the data, such as in kernel convolution, or Getis & Franklin's local point pattern analysis. In the current approach, only one point is evaluated by a model at a time and only the chosen number of near neighbors need to be considered, which means very large datasets can be processed without requiring exorbitant computing resources.

Model specification and training

Input sequences for both clustered and non-clustered points were randomly selected from each simulated cluster scenario dataset (two thousand points of each type, or as many as were available) and pooled according to the clustering type. From this initial pair of training pools three distinct subset of entries, and their matching classification labels, were randomly drawn to create the final training, validation, and testing datasets. An equal number of clustered and non-clustered input sequences were selected for all sets. The models described here were trained on 500,000 input sequences, validated on 100,000 input sequences, and tested on 100,000 input sequences.

Training was performed in Keras using the TensorFlow back-end (version 1.8.0) over 100 epochs with a batch size of 32. The ADAM optimizer was used with an initial model learning rate of 0.001 which reduced with each new epoch and loss determined by binary cross-entropy. The model scores each input sequence in the range of 0.0 to 1.0, representing the likelihood of that sequence originated from a point with the 'clustered' label. A threshold was applied to convert scores into labels of either 0 ('non-clustered' label for scores ≤ 0.5) or 1 ('clustered' label for scores > 0.5). For performance evaluation, the model-assigned labels were compared to the ground-truth labels for all points in the simulated data. After training the model was exported to disk along with training, validation, and testing performance tracking across the epochs. The performance of the final model was evaluated on the testing data to determine scores for precision, recall, and F1 score (a combined precision and recall score) and to generate confusion matrices. A further estimate of a trained model's performance was calculated using 10-fold cross-validation to check the performance stability over multiple subsets of the testing data.

Novel data were prepared as described and the resulting input used with trained models to generate a list of classifications matching every point from the original image. The classifications denote if a point is either clustered or not. Processing can be stopped at this stage, if only this amount of information is required. Otherwise the classified data can be used for the subsequent post-evaluation cluster segmentation stage or as input to any other post-processing pipeline for further annotation.

Post-evaluation cluster segmentation

Individual clusters were extracted from the set of each points' *xy* coordinates, classification label, near-neighbor distances, and near-neighbors' unique identifiers. From the set of points, those points which were labelled as 'clustered' were sorted according to descending clustering score and each assigned a unique starting 'Cluster ID'. Beginning with the most clustered point, its list of near-neighbor points was retrieved. If the nearest neighboring point was also labelled as clustered, then it was considered to be in the same cluster. Consecutive neighboring points were considered in turn and grouped into the same cluster as the origin point if they were also labelled as clustered points. This process stopped when the first non-clustered neighboring point was reached. If any of the nearby-clustered points have already been assigned a Cluster ID, then all points adopt that Cluster ID (if there are several other Cluster IDs then the lowest Cluster ID is adopted by those points. All other points with the competing Cluster IDs are also reassigned in this manner). Otherwise all points keep the original Cluster ID and the process is repeated with another clustered point until all clustered points have been assigned a Cluster ID.

This method therefore utilizes all the labelling information from the model: clustered points are used to initiate the growth of a new cluster and non-clustered points used to halt the process. However, this approach will only succeed while there are non-clustered points present to indicate when a cluster should stop acquiring new points. In cases where a large majority of the points are clustered there are two approaches that can be used to segment clusters which are separated by empty space rather than non-clustered points. First, in cases where all the nearest points (as many as were measured in the pre-processing step) were labelled as 'clustered' by the model, an additional check can be triggered, which considers the spatial arrangement of these points. Here the mean distance to the nearest three points is taken and compared against the mean as more consecutive nearby points are included. If the mean distance increases by more than 5 times the standard deviation of the mean, then the cluster is 'split' at the previous point and all earlier points assigned the same Cluster ID. Once all clustered points have been assigned a Cluster ID, each Cluster ID is checked to ensure it contains at least three points. Points are relabeled as non-clustered if their Cluster ID fails this test. Next, each set of like-clustered points (sharing the same Cluster ID) was used to fit an encapsulating cluster shape.

Post-segmentation shape fitting

A set of points has no intrinsic area, but a polygon can be constructed which contains the set of points and approximates the intuitive shape of the spatial distribution of those points, including spurs and (with additional polygons) holes and other features. Such a polygon can be created by several methods including bounding box, convex- and alpha-hulls, however these methods cannot always produce a shape which satisfactorily describes the distribution of the points. The method used here involves dilating each point in the set by a certain radius, then taking the union of all the overlapping discs from each dilated point to form the cluster polygon. In this fashion a cluster shape can be constructed which approximates the underlying distribution of points and is not limited to circular clusters; points laid out in a line will produce an elongated polygon (Supplementary Figure 1). The selection of the dilation radius is an important parameter in this process, for if the dilation radius is too small then the shapes formed from points may not overlap and the 'cluster' fragments into disconnected polygons. If the dilation radius is too large, then the resultant shape contains all the points but is a poor representation of the spatial distribution of those points. A suitable radius can be estimated from the ratio of the bounding-box area of the set of points to the number of points in the set. In this way, the dilation radius adapts to the area over which the points are spread and the number of points which need to be enclosed. To reduce the extent to which the resultant shape's boundary falls beyond the 'outer' points, the cluster polygon can also be eroded by a fraction of the dilation radius to yield a final shape which contains all the prescribed points in a relatively free conformation. This method may generate multiple polygons if, for example, in the case where two clusters were close enough to be considered as one by the segmentation process but are separated by a distance greater than the dilation radius used to create the cluster outline. In these cases, polygons containing more than a specified number of points (again here we used three or more points) can be considered as separate clusters and their constituent points given separate new unique Cluster IDs. Polygons containing fewer points are not considered to be clusters and their points can be stripped of the Cluster ID and their cluster label re-assigned to 'non-clustered'.

The points in our synthetic clusters are distributed about a cluster center, up to a maximum distance from the center, and the distribution of points within the cluster is random. Therefore, the cluster shape which is constructed by this stage will follow the distribution of the points and not the original maximum radius (of which it has no knowledge). For a sufficiently dense cluster, however, the resulting shape construction from so many overlapping point-dilations will likely approximate the original circular specification.

This method may also generate polygons containing holes; these holes can either be preserved or removed to close the hole. When closing holes, it is possible to specify a relative size threshold below which holes are closed and above which the holes are preserved in order to better represent, for

example, ring-shaped clusters. Here we removed holes which were smaller than half of the dilation radius used to construct the cluster shape.

The final cluster shape can be assessed for various shape descriptors such as the cluster area, perimeter length, and circularity ratio. As the number of points in the cluster is known further metrics such as the density of points within the cluster can be determined. As the entire original image is processed, thousands of clusters can be assessed and slight differences in cluster distributions relative to particular areas of the cell may become apparent and these can be further examined using targeted regions of interest.

Comparison to Getis & Franklin's Local Point Pattern Analysis

The simulated datasets used to assess model performance were also used to evaluate the performance of different cluster analysis methods. A stalwart method is that of Getis & Franklin: their local point-pattern analysis (G&F LPPA) is a spatially restricted version of the original Ripley's K Function and Besag's H Function, in that it only considers the density of points at a single fixed radius. Each point acquires a value indicating if the local point density (within the fixed radius) is greater or less than the expected point density; this value is effectively a measure of the point's clustering behavior at the specified spatial scale. This value can then be subjected to a threshold to classify points as clustered (above threshold) or non-clustered (below threshold). However, the specification of the radius and threshold are very much dependent on the underlying data and are sensitive to multiple scales of clustering and inhomogeneities (for example a very large dense cluster of points somewhere in the data can skew the assessment of the 'expected point density' considerably). As the simulated datasets contain only a single type of cluster which is evenly distributed through the 'cell area' a G&F LPPA analysis should be appropriate for our simulated datasets. The analysis was favored by using a radius which was matched to the expected cluster radius for each specific dataset. A threshold for each dataset was chosen by first analyzing five datasets of the equivalent number of points as the original, but with xy coordinates spatially randomized within the cell-shape. The 99.5 percentile value for all $L(r)$ values from all five randomized images was used as the threshold value for the original dataset.

Comparison to Bayesian Inference method

As this method can be computationally intensive for datasets with more than approximately 25,000 points, data were analyzed from a 3 × 3 µm region in the center of each simulated dataset. Analysis was performed in R (v3.6.2 in RStudio v1.1.463) using a version of the original published script (and using its priors) which had been modified to permit parallel processing and for better memory management.

Comparison to DBSCAN

The implementation of DBSCAN from PALMsiever[37] was used to analyze simulated and experimental data in MATLAB (2017b). The 'minPts' (minimum points) parameter was set to 3 to match the minimum requirement for a cluster used elsewhere. The 'eps' (epsilon) parameter was determined empirically to deliver the best result or was set to 'automatic' in which case the value was a function of the overall point density.

Comparison to SR-Tesseler

The latest publicly available version of SR-Tesseler (v1.0.0.1) was downloaded from http://www.iins.u-bordeaux.fr/team-sibarita-SR-Tesseler. The included dataset 'gluA1_mEOS2.csv' was used for comparisons between methods. As this version of SR-Tesseler does not have the option to export coordinate data, fluorophore blinking correction was performed in ThunderSTORM (25 nm distance, 20 frames off-time) to allow the same coordinate data to be used across different clustering software. The remainder of the parameter selection (object and cluster construction) was guided by the online user manual; limitations on the minimum or maximum allowable cluster areas were

removed. The minimum number of points required to form a cluster was set to 3 to match those used by other methods and no maximum was specified. The 'density factor' parameter was selected for the best-performing value in cases where the expected clustering was known. Comparisons to other methods were performed subjectively on exported snapshots from the 'viewer' or on the exported summary clustering statistics as per-point data was not available with this version of the software.

Cell Culture & Transfections

T-like cells Jurkat E6.1 (ECACC 88042803) were maintained in RPMI supplemented with 10% fetal bovine serum, and L-glutamine. These cells were transfected using a Lonza Nucleofector II device by electroporating 1×10^6 cells with 2 µg endotoxin-free plasmid DNA in complete Nucleofector Solution V with program 'X-005' according to the manufacturer's instructions.

Human foreskin fibroblast cells HFFF2 (ECACC 86031405) were maintained in DMEM (High Glucose) supplemented with 10% fetal bovine serum, and L-glutamine.

Sample preparation and imaging

For microtubule images, HFF fibroblasts were seeded onto glass-bottomed chamber slides at 5×10^3 cells/cm^2 and allowed 1-2 days to adhere and spread. Microtubules were first washed with warm PHEM Buffer (80 mM PIPES pH 7.4, 25 mM HEPES, 5 mM EGTA, 2 mM $MgCl_2$) and then fixed in warm PHEM Buffer supplemented with 3% paraformaldehyde and 0.25% glutaraldehyde for 12-15 minutes at 37°C. Fixed cells were washed in Tris-buffered saline (TBS) and permeabilized in TBS with 0.01% (w/v) lysolecithin (Sigma L4129) for ten minutes. Permeabilized cells were quenched with 300 mM glycine in TBS and then blocked with 2% BSA, 0.2% fish-skin gelatin in TBS. Monoclonal anti-alpha tubulin antibody (Clone DM1A, eBioscience) was used at 1:400 (in blocking buffer) overnight at 4°C. After washing, goat anti-mouse antibody conjugated to Alexa Fluor 647 (ThermoFisher Scientific A-21237) was then applied for 1 hour at room temperature. After more washes the sample was placed in a pyranose oxidase-based oxygen-scavenging dSTORM buffer prior to image acquisition.

For LAT images, Jurkat E6.1 cells expressing LAT-mEos3.2 were introduced to anti-CD3 (2 µg/ml) and anti-CD28 (5 µg/ml) coated glass-bottomed chamber slides (at 50×10^3 cells/cm^2 in warm HBSS) and incubated at 37°C for 5 minutes to allow for synapse formation. The chamber wells were gently washed with warm HBSS and then fixed in 3% paraformaldehyde in phosphate-buffered saline (PBS) for 20 minutes at 37°C. Fixed cells were washed in PBS, quenched with 300 mM glycine in PBS, washed again, and used immediately for PALM imaging.

PALM images were acquired on the same Nikon NSTORM system used for dSTORM imaging. Samples were continuously illuminated with 561 nm laser light at approximately 2 kW/cm^2 and 405 nm laser light (to induce photo-conversion) at approximately 2 W/cm^2. Images were recorded at 40 ms per frame for 5,000 to 15,000 frames. PALM images were reconstructed and post-processed in ThunderSTORM.

References

37. Pengo, T., Holden, S. J. & Manley, S. PALMsiever: a tool to turn raw data into results for single-molecule localization microscopy. *Bioinforma. Oxf. Engl.* **31**, 797–798 (2015).

Supplementary Table 1

Ratio of point density inside and outside of clusters for combinations of simulated cluster parameters. PpMS = Point density (per μm^2), PpC = Points per Cluster, Max. Radius = maximum distance a clustered point may be from the center of a cluster, n/a = not applicable; scenarios where the clustered points are either all or none of the population. Values in bold represent 'viable' scenarios (to use in training models) where the density ratio is between 1.5× and 100×, except for 100% clustering which was also accepted. Scenarios with no clustering were generated at each point density for evaluation datasets.

PpMS	PpC	Max. Radius	Percent of Points Clustered											
			0	5	10	20	30	40	50	60	70	80	90	100
50	10	10	-	670.1	707.2	795.5	909.0	1060.4	1272.2	1590.0	2119.7	3179.1	6357.2	-
50	10	20	-	167.5	176.7	198.7	226.9	264.6	317.3	396.4	528.2	791.8	1582.5	-
50	10	30	-	**74.4**	**78.5**	**88.2**	100.6	117.2	140.5	175.3	233.5	349.7	698.4	-
50	10	40	-	**41.8**	**44.1**	**49.5**	**56.4**	**65.6**	**78.6**	**98.0**	130.3	194.9	388.9	-
50	10	50	-	**26.8**	**28.2**	**31.6**	**35.9**	**41.8**	**49.9**	**62.2**	**82.5**	123.3	245.6	-
50	10	60	-	**18.6**	**19.5**	**21.9**	**24.8**	**28.8**	**34.4**	**42.7**	**56.6**	**84.4**	167.8	-
50	10	70	-	**13.6**	**14.3**	**16.0**	**18.1**	**21.0**	**25.0**	**31.0**	**41.0**	**61.0**	120.9	-
50	10	80	-	**10.4**	**10.9**	**12.2**	**13.8**	**15.9**	**18.9**	**23.4**	**30.8**	**45.7**	**90.5**	-
50	10	90	-	**8.2**	**8.6**	**9.6**	**10.8**	**12.4**	**14.7**	**18.1**	**23.9**	**35.3**	**69.6**	-
50	10	100	-	**6.6**	**7.0**	**7.7**	**8.7**	**9.9**	**11.7**	**14.4**	**18.9**	**27.8**	**54.7**	-
100	20	10	-	670.1	707.2	795.5	909.0	1060.4	1272.2	1590.0	2119.7	3179.1	6357.2	-
100	20	20	-	167.5	176.7	198.7	226.9	264.6	317.3	396.4	528.2	791.8	1582.5	-
100	20	30	-	**74.4**	**78.5**	**88.2**	100.6	117.2	140.5	175.3	233.5	349.7	698.4	-
100	20	40	-	**41.8**	**44.1**	**49.5**	**56.4**	**65.6**	**78.6**	**98.0**	130.3	194.9	388.9	-
100	20	50	-	**26.8**	**28.2**	**31.6**	**35.9**	**41.8**	**49.9**	**62.2**	**82.5**	123.3	245.6	-
100	20	60	-	**18.6**	**19.5**	**21.9**	**24.8**	**28.8**	**34.4**	**42.7**	**56.6**	**84.4**	167.8	-
100	20	70	-	**13.6**	**14.3**	**16.0**	**18.1**	**21.0**	**25.0**	**31.0**	**41.0**	**61.0**	120.9	-
100	20	80	-	**10.4**	**10.9**	**12.2**	**13.8**	**15.9**	**18.9**	**23.4**	**30.8**	**45.7**	**90.5**	-
100	20	90	-	**8.2**	**8.6**	**9.6**	**10.8**	**12.4**	**14.7**	**18.1**	**23.9**	**35.3**	**69.6**	-
100	20	100	-	**6.6**	**7.0**	**7.7**	**8.7**	**9.9**	**11.7**	**14.4**	**18.9**	**27.8**	**54.7**	-
300	100	10	-	1116.8	1178.8	1326.0	1515.3	1767.7	2121.1	2651.1	3534.4	5301.2	10601.3	-
300	100	20	-	279.2	294.6	331.3	378.5	441.4	529.5	661.6	881.9	1322.3	2643.6	-
300	100	30	-	124.0	130.9	147.1	168.0	195.8	234.8	293.2	390.6	585.5	1169.9	-
300	100	40	-	**69.8**	**73.6**	**82.6**	**94.3**	109.9	131.6	164.3	218.7	327.6	654.1	-
300	100	50	-	**44.6**	**47.0**	**52.8**	**60.2**	**70.1**	**83.9**	104.6	139.1	208.2	415.4	-
300	100	60	-	**31.0**	**32.6**	**36.6**	**41.7**	**48.5**	**57.9**	**72.2**	**95.9**	143.4	285.7	-
300	100	70	-	**22.7**	**23.9**	**26.8**	**30.5**	**35.4**	**42.3**	**52.6**	**69.8**	104.3	207.5	-
300	100	80	-	**17.4**	**18.3**	**20.5**	**23.3**	**27.0**	**32.2**	**39.9**	**52.9**	**78.9**	156.8	-
300	100	90	-	**13.7**	**14.4**	**16.1**	**18.3**	**21.2**	**25.2**	**31.2**	**41.3**	**61.5**	122.0	-
300	100	100	-	**11.1**	**11.7**	**13.0**	**14.7**	**17.0**	**20.2**	**25.0**	**33.0**	**49.1**	**97.1**	-
500	80	10	-	536.0	565.8	636.4	727.1	848.2	1017.6	1271.7	1695.3	2542.5	5084.0	-
500	80	20	-	134.0	141.4	158.9	181.5	211.5	253.6	316.8	422.1	632.6	1264.2	-
500	80	30	-	**59.5**	**62.8**	**70.5**	**80.4**	**93.6**	112.2	140.0	186.3	278.9	556.9	-
500	80	40	-	**33.5**	**35.3**	**39.5**	**45.0**	**52.4**	**62.7**	**78.1**	103.8	155.2	309.3	-
500	80	50	-	**21.4**	**22.5**	**25.2**	**28.7**	**33.3**	**39.7**	**49.4**	**65.6**	**97.9**	194.7	-
500	80	60	-	**14.8**	**15.6**	**17.4**	**19.8**	**22.9**	**27.3**	**33.9**	**44.8**	**66.7**	132.5	-
500	80	70	-	**10.9**	**11.4**	**12.7**	**14.4**	**16.7**	**19.8**	**24.5**	**32.3**	**48.0**	**94.9**	-
500	80	80	-	**8.3**	**8.7**	**9.7**	**10.9**	**12.6**	**14.9**	**18.4**	**24.2**	**35.8**	**70.6**	-
500	80	90	-	**6.6**	**6.9**	**7.6**	**8.6**	**9.8**	**11.6**	**14.2**	**18.6**	**27.4**	**53.9**	-
500	80	100	-	**5.3**	**5.5**	**6.1**	**6.8**	**7.8**	**9.2**	**11.2**	**14.6**	**21.5**	**41.9**	-

Supplementary Table 2

Summary statistics for Csk data shown in Supplementary Figure 11a-f.

Csk - Percent clustering	Number of cells	Minimum	25% Percentile	Median	75% Percentile	Maximum
Naïve - Glass	85	40.41	48.33	53.21	59.69	86.9
Naïve - CD3 + ICAM-1	71	46.98	53.14	61.15	64.39	74.72
Pre-stimulated - Glass	30	38.7	49.82	54.01	59.47	69.3
Pre-stimulated - CD3 + ICAM-1	19	48.17	54.82	59.12	67.92	73.3

Csk - Clusters per um^2	Number of cells	Minimum	25% Percentile	Median	75% Percentile	Maximum
Naïve - Glass	85	1.179	3.153	4.845	7.378	15.06
Naïve - CD3 + ICAM-1	71	2.806	6.747	8.8	10.75	17
Pre-stimulated - Glass	30	1.439	5.132	6.773	8.372	13.88
Pre-stimulated - CD3 + ICAM-1	19	3.608	11.03	14.37	18.57	22.25

Csk - Points per um^2	Number of cells	Minimum	25% Percentile	Median	75% Percentile	Maximum
Naïve - Glass	85	46.85	86.39	114.5	165.5	442.9
Naïve - CD3 + ICAM-1	71	97.19	165.6	210.3	286.8	526.6
Pre-stimulated - Glass	30	33.18	155.5	224	343.6	706.3
Pre-stimulated - CD3 + ICAM-1	19	70.5	364.1	513.7	721	846.6

Csk - Points per cluster	Number of clusters	Minimum	25% Percentile	Median	75% Percentile	Maximum
Naïve - Glass	23672	2	6	9	14	365
Naïve - CD3 + ICAM-1	64809	2	7	11	18	569
Pre-stimulated - Glass	34954	2	7	12	22	398
Pre-stimulated - CD3 + ICAM-1	58476	2	8	14	27	543

Csk - Area per cluster	Number of clusters	Minimum	25% Percentile	Median	75% Percentile	Maximum
Naïve - Glass	23672	19.87	1310	2322	4522	728563
Naïve - CD3 + ICAM-1	64809	10.2	1261	2144	3770	174256
Pre-stimulated - Glass	34954	24.51	1632	3093	6154	153784
Pre-stimulated - CD3 + ICAM-1	58476	8.633	1030	1920	3655	77267

Csk - Density per cluster	Number of clusters	Minimum	25% Percentile	Median	75% Percentile	Maximum
Naïve - Glass	23672	122	2206	3921	6558	201321
Naïve - CD3 + ICAM-1	64809	224	3446	5333	8028	196043
Pre-stimulated - Glass	34954	141	2517	3939	6243	131833
Pre-stimulated - CD3 + ICAM-1	58476	291	5517	7876	11173	251605

Supplementary Table 3

Summary statistics for PAG data shown in Supplementary Figure 11g-l.

PAG - Percent clustering	Number of cells	Minimum	25% Percentile	Median	75% Percentile	Maximum
Naïve - Glass	53	40.48	47.38	50.45	54.45	69.65
Naïve - CD3 + ICAM-1	65	41.79	50.36	54.57	59.09	70.63
Pre-stimulated - Glass	14	57.69	59.77	64.65	68.77	73.68
Pre-stimulated - CD3 + ICAM-1	18	56.96	60.24	63.99	66.94	70.86

PAG - Clusters per um^2	Number of cells	Minimum	25% Percentile	Median	75% Percentile	Maximum
Naïve - Glass	53	0.6649	5.034	10.19	16.07	23.4
Naïve - CD3 + ICAM-1	65	0.6848	7.074	13.89	21.32	27.05
Pre-stimulated - Glass	14	0.2951	0.5227	1.205	1.599	1.767
Pre-stimulated - CD3 + ICAM-1	18	0.5368	0.7756	0.9935	1.103	1.604

PAG - Points per um^2	Number of cells	Minimum	25% Percentile	Median	75% Percentile	Maximum
Naïve - Glass	53	18.32	169.6	373.1	705.6	1208
Naïve - CD3 + ICAM-1	65	15.59	271.6	460	685.7	971.3
Pre-stimulated - Glass	14	6.77	12.81	42.84	76.44	97.89
Pre-stimulated - CD3 + ICAM-1	18	10.59	19.9	26.69	32.46	54.26

PAG - Points per cluster	Number of clusters	Minimum	25% Percentile	Median	75% Percentile	Maximum
Naïve - Glass	31813	2	6	10	18	807
Naïve - CD3 + ICAM-1	98455	2	6	10	18	694
Pre-stimulated - Glass	3156	3	7	14	32	435
Pre-stimulated - CD3 + ICAM-1	4309	2	6	11	20	267

PAG - Area per cluster	Number of clusters	Minimum	25% Percentile	Median	75% Percentile	Maximum
Naïve - Glass	31813	19.76	840.9	1551	3159	284412
Naïve - CD3 + ICAM-1	98455	1.32	766	1359	2522	140336
Pre-stimulated - Glass	3156	243	8597	19504	41690	194462
Pre-stimulated - CD3 + ICAM-1	4309	143.7	8054	15669	30041	183187

PAG - Density per cluster	Number of clusters	Minimum	25% Percentile	Median	75% Percentile	Maximum
Naïve - Glass	31813	128	4383	6828	10335	182152
Naïve - CD3 + ICAM-1	98455	106	5635	8138	11645	2272812
Pre-stimulated - Glass	3156	60	568	866.5	1320	12345
Pre-stimulated - CD3 + ICAM-1	4309	59	509	782	1214	34800

Supplementary Note 1

Simulated Data Conditions

The list of clustering parameters used to generate cluster scenarios is detailed below.

Model training, validation, and testing:

- Total replicates per scenario: 1
- Points per cluster: 5, 10, 20, 30, 40, 50, 60, 70, 80, 90
- Radii (maximum, nm): 5, 10, 15, 25, 40, 50, 75, 100
- Percentage points in clusters: 5, 10, 20, 30, 40, 50, 60, 70, 80, 90, 100
- Points per μm^2: 5, 10, 50, 100, 300, 500
- Limits: $1 \leq$ Clusters per $\mu m^2 \leq 5$ and $1.5 <$ Density Ratio < 100
- Total clustering scenarios: 711 (711 images)

Model performance evaluation:

Clustered data:

- Total replicates per scenario: 10
- Percentage points in clusters: 5, 10, 20, 30, 40, 50, 60, 70, 80, 90, 100
- Limits: $0.25 \leq$ Clusters per $\mu m^2 \leq 5.0$
- 50 Points per μm^2, 10 Points per cluster:
    - Radii (maximum, nm): 10, 20, 30, 40, 50, 60, 70, 80, 90, 100
    - Total clustering scenarios: 110 (1100 images)
- 100 Points per μm^2, 20 Points per cluster:
    - Radii (maximum, nm): 5, 10, 20, 30, 40, 50, 60, 70, 80, 90, 100
    - Total clustering scenarios: 121(1210 images)
- 300 Points per μm^2, 100 Points per cluster:
    - Radii (maximum, nm): 10, 20, 30, 40, 50, 60, 70, 80, 90, 100
    - Total clustering scenarios: 110 (1100 images)
- 500 Points per μm^2, 80 Points per cluster
    - Radii (maximum, nm): 5, 10, 20, 30, 40, 50, 60, 70, 80, 90, 100
    - Total clustering scenarios: 121 (1210 images)

Spatially random (no clusters) data:

- Total replicates per scenario: 10
- Points per cluster: 0
- Radii (maximum, nm): 0
- Percentage points in clusters: 0
- Points per μm^2: 10, 50, 100, 300, 500, 3000
- Total CSR scenarios: 6 (60 images)

Supplementary Note 2

Training times and resource requirements

Data processing times and storage space consumption required for model training. The following times are indicative of performance on 10 cores of an Intel Core i7-4930K CPU with 32 MB RAM using locally attached storage and GPU-assisted training (NVIDIA GTX 750 Ti). Processing time will vary depending on overall computer specification as well as the size and location of data, number of nearest neighbors, number of training, validation, and testing samples, the type and arrangement of layers in a model, and the number of training epochs. Storage requirements will vary depending on the size of the input data and the number of nearest neighbors to which distances are measured.

- Stage 0 - Generation of 711 training images (for 711 scenarios) took 48 minutes and required 4.3 GiB storage.
- Stage 1 - Distance measurements for 81,388,567 points (from 711 training images) to their 100 nearest neighbors took 11.9 hours and required 12.3 GiB storage.
- Stage 2 - Creation of training corpus randomly drawn from Stage 1 data and comprising 500,000 training samples, 500,000 validation samples, and 100,000 testing samples took 8.5 minutes and required 550 MiB storage.
- Stage 3 - Model training, testing, and validation.
    - With 12 layers (equivalent to 07VEJJ) and 100 epochs: 8.3 hours
    - With 4 layers (equivalent to XPILJZ) and 100 epochs: 2.4 hours
