# Supplementary material for: Machine learning for cluster analysis of localization microscopy data
Source: Nat Commun. 2020 Mar 20;11:1493. doi: 10.1038/s41467-020-15293-x (PMC7083906; doi:10.1038/s41467-020-15293-x)
Supplement: Supplementary file 3 — Reporting Summary [file 41467_2020_15293_MOESM3_ESM.pdf]

nature research

Corresponding author(s): OWEN, Dylan M.

Last updated by author(s): Feb 20, 2020

Reporting Summary

Nature Research wishes to improve the reproducibility of the work that we publish. This form provides structure for consistency and transparency in reporting. For further information on Nature Research policies, see Authors & Referees and the Editorial Policy Checklist.

Statistics

For all statistical analyses, confirm that the following items are present in the figure legend, table legend, main text, or Methods section.

n/a	Confirmed	
☐	☒	The exact sample size (*n*) for each experimental group/condition, given as a discrete number and unit of measurement
☐	☒	A statement on whether measurements were taken from distinct samples or whether the same sample was measured repeatedly
☐	☒	The statistical test(s) used AND whether they are one- or two-sided<br>*Only common tests should be described solely by name; describe more complex techniques in the Methods section.*
☐	☒	A description of all covariates tested
☐	☒	A description of any assumptions or corrections, such as tests of normality and adjustment for multiple comparisons
☐	☒	A full description of the statistical parameters including central tendency (e.g. means) or other basic estimates (e.g. regression coefficient) AND variation (e.g. standard deviation) or associated estimates of uncertainty (e.g. confidence intervals)
☐	☒	For null hypothesis testing, the test statistic (e.g. *F*, *t*, *r*) with confidence intervals, effect sizes, degrees of freedom and *P* value noted<br>*Give P values as exact values whenever suitable.*
☐	☒	For Bayesian analysis, information on the choice of priors and Markov chain Monte Carlo settings
☒	☐	For hierarchical and complex designs, identification of the appropriate level for tests and full reporting of outcomes
☐	☒	Estimates of effect sizes (e.g. Cohen's *d*, Pearson's *r*), indicating how they were calculated

Our web collection on statistics for biologists contains articles on many of the points above.

Software and code

Policy information about availability of computer code

Data collection	Nikon NIS Elements (v4), Python (3.7)
Data analysis	ThunderSTORM (dev-2015-10-03-b1), Python (3.7), custom Python scripts (https://gitlab.com/quokka79/caml), Graphpad Prism (8.3.1), R (3.6.2) and RStudio (1.1.463), PALMsiever (2014-09-04) in MATLAB (2017b), ST-Tesseler (v1.0.0.1)

For manuscripts utilizing custom algorithms or software that are central to the research but not yet described in published literature, software must be made available to editors/reviewers. We strongly encourage code deposition in a community repository (e.g. GitHub). See the Nature Research guidelines for submitting code & software for further information.

Data

Policy information about availability of data

All manuscripts must include a data availability statement. This statement should provide the following information, where applicable:

- Accession codes, unique identifiers, or web links for publicly available datasets
- A list of figures that have associated raw data
- A description of any restrictions on data availability

Custom code used to generate and analyse data is available at https://gitlab.com/quokka79/caml. Model training, validation, and testing data available from https://osf.io/xa4zj/.

Field-specific reporting

Please select the one below that is the best fit for your research. If you are not sure, read the appropriate sections before making your selection.

☒ Life sciences ☐ Behavioural & social sciences ☐ Ecological, evolutionary & environmental sciences

For a reference copy of the document with all sections, see nature.com/documents/nr-reporting-summary-flat.pdf

Life sciences study design

All studies must disclose on these points even when the disclosure is negative.

Sample size	Sample-size calculations were not performed. Data on primary T cells were obtained from three donors as this is what was available to us at the time. A minimum of ten regions (enclosing cell synapses) were required for each condition; on average 47 regions per condition were analysed.
Data exclusions	dSTORM data were excluded from further analysis if the reconstructed images could not be corrected for sample drift, e.g. due to lack of features to allow RCC drift-correction.
Replication	T cell data was reproduced from three different donors. Neural-network models were rebuilt using the same layer configurations on different training data and compared for accuracy and all models were subjected to ten-fold cross-validation. Replication of the programming environment and analysis process was conducted by an inexperienced volunteer. Software pseudo-random number generators were initialized with a fixed seed.
Randomization	Cells were selected for imaging from random starting locations within the sample. Blood donors were selected at random.
Blinding	Blinding was not performed as selection bias was not considered relevant to this study.

Reporting for specific materials, systems and methods

We require information from authors about some types of materials, experimental systems and methods used in many studies. Here, indicate whether each material, system or method listed is relevant to your study. If you are not sure if a list item applies to your research, read the appropriate section before selecting a response.

Materials & experimental systems

n/a	Involved in the study	
☐	☒	Antibodies
☐	☒	Eukaryotic cell lines
☒	☐	Palaeontology
☒	☐	Animals and other organisms
☒	☐	Human research participants
☒	☐	Clinical data

Methods

n/a	Involved in the study	
☒	☐	ChIP-seq
☒	☐	Flow cytometry
☒	☐	MRI-based neuroimaging

Antibodies

Antibodies used	anti-CD3 mAb ThermoFisher Scientific 16-0037-81, clone OKT3), anti-CD28 mAb (ThermoFisher Scientific 16-0289-85, Clone CD28.2), anti-Csk pAb (Santa Cruz sc-286), anti-PAG pAb (Abcam ab14989), anti-alpha-Tubulin mAb (eBioscience DM1A) anti-mouse pAb (ThermoFisher Scientific A-21237, Alexa Fluor 647 conjguated)
Validation	anti-CD3 (verified by relative expression by manufacturer), anti-CD28 (verified by functional assay by manufacturer), anti-Csk (validation by western blot and immunofluorescence by manufacturer), anti-PAG (validation by western blot and immunofluorescence by manufacturer), anti-alpha-Tubulin (validation by western blot and immunofluorescence by manufacturer), anti-mouse-IgG (validation by western blot and immunofluorescence by manufacturer).

Eukaryotic cell lines

Policy information about cell lines

Cell line source(s)	Jurkat E6.1 (ECACC 88042803), Human foreskin fibroblast cells HFFF2 (ECACC 86031405)
Authentication	Not authenticated
Mycoplasma contamination	Cell lines tested negative for mycoplasma contamination (by qPCR, Eurofins Genomics)

Commonly misidentified lines
(See ICLAC register)

None used.
